# Optimization of fermentation conditions for enhanced acetylcholine and biomass production of *Lactiplantibacillus plantarum* AM2 using the Taguchi approach

**DOI:** 10.1186/s12866-025-04017-0

**Published:** 2025-05-22

**Authors:** Walid A. Lotfy, Amira M. Ali, Heba M. Abdou, Khaled M. Ghanem

**Affiliations:** 1 Department of Microbiology, Faculty of Dentistry, Pharos University in Alexandria, Alexandria, Egypt; 2 Department of Botany and Microbiology, Faculty of Science, Alexandria University, Alexandria, Egypt; 3 Department of Zoology, Faculty of Science, Alexandria University, Alexandria, Egypt

**Keywords:** Acetylcholine, Biomass, *Lactiplantibacillus plantarum*, Taguchi

## Abstract

This study aimed to optimize the fermentation conditions and medium composition for maximum acetylcholine (ACh) and biomass production by *Lactiplantibacillus plantarum* AM2 using the Taguchi array design, which enables efficient identification of influential variables through minimal experimental runs. Seven key factors were evaluated: beef extract, peptone, yeast extract, glucose, pH, agitation rate, and inoculation size. The optimization process identified the most significant variables influencing ACh and biomass production, with beef extract and peptone being critical for ACh synthesis, while inoculation size was a critical determinant of biomass yield. The optimal conditions for ACh production were determined as beef extract (11 g/l), peptone (40 g/l), yeast extract (5 g/l), glucose (20 g/l), pH 5.7, no agitation, and 1% (v/v) inoculation size, resulting in a predicted ACh concentration of 490.83 pg/ml and an experimental value of 495.8 pg/ml. For biomass production, the optimal conditions were beef extract (8 g/l), peptone (10 g/l), yeast extract (20 g/l), glucose (35 g/l), pH 6.6, agitation at 150 rpm, and 4% (v/v) inoculation size, yielding a predicted biomass of 20.58 g/l and an experimental value of 21.3 g/l. The optimized conditions significantly improved ACh production (6.32-fold) and biomass production (4.56-fold) compared to basal conditions. These findings highlight the efficiency of the Taguchi approach in enhancing the production of ACh and biomass, providing insights into the functional niche of *Lactiplantibacillus plantarum* AM2 for potential industrial applications and its use in a symbiotic form.

## Introduction

The major bacterial genera used in probiotic products can be classified into two groups: (i) lactic acid-producing bacteria, including *Bifidobacterium*, *Lactiplantibacillus*, and *Streptococcus*; and (ii) non-lactic acid-producing bacteria, including *Bacillus* and *Propionibacterium* [1]. *Lactiplantibacillus plantarum*, belonging to the genus *Lactiplantibacillus*, is part of the facultatively heterofermentative group of lactobacilli. This species exhibits remarkable diversity and adaptability, thriving in a wide range of ecological niches.

*Lactiplantibacillus* are fastidious and have strain-specific nutrient and environmental requirements, necessitating a rich medium and optimal conditions for robust growth of each strain.Their viability is greatly influenced by fermentation conditions, including pH, medium composition, oxygen levels, and inoculum size [2]. Moreover, nutritional requirements play a vital role in the growth and development of probiotic strains [3]. Carbon and nitrogen sources, particularly amino acids, are essential for supporting the metabolic functions of Lactobacilli and *Bifidobacteria* strains [3]. Alterations in raw materials can have a substantial effect on growth and efficiency [3]. While complex substances such as milk and yeast extract serve as protein sources, simpler ingredients like salts and simple carbohydrates are frequently utilized as carbon sources [4]. The nutritional needs of *Lactiplantibacillus* are often influenced by the complexity of their autotrophic and nutrient demands associated with their environment [5]. For instance, *Lactiplantibacillus plantarum* has a lower number of autotrophs as it is frequently isolated from plant origins [6]. On the other hand, *Lactobacillus johnsonii*, which is obtained from the upper gastrointestinal tract of humans, exhibits greater self-sufficiency in biosynthesis and thrives in an environment rich in resources like small peptides, amino acids, and polysaccharides [7]. Thus, developing a distinctive fermentation solution for *Lactiplantibacillus* requirements enhances the production of high-quality end products [8–14]. *Lactiplantibacillus plantarum*, a member of the lactic acid-producing bacteria, is widely recognized for its probiotic potential and versatility across diverse ecological niches. Due to its fastidious growth requirements and strain-specific nutritional demands, optimizing fermentation conditions is essential to enhance its metabolic activity and biomass yield. Factors such as carbon and nitrogen sources, pH, oxygen levels, and inoculum size are known to significantly influence its growth and metabolite production.

Among its potential bioactive metabolites, the neurotransmitter acetylcholine of *Lactiplantibacillus plantarum* has gained attention for its crucial role in the central and peripheral nervous systems, particularly in cognitive functions such as memory, attention, and learning [15]. Acetylcholine is synthesized from choline, a precursor that is derived from the diet and can also be produced endogenously in the liver. In the human brain, acetylcholine is produced by cholinergic neurons and is involved in synaptic transmission within the hippocampus, cortex, and other regions associated with memory and cognition [15]. The vital role of acetylcholine in neuroplasticity and synaptic activity makes it a key target in therapeutic approaches for neurodegenerative diseases like Alzheimer’s disease, where its levels are significantly reduced [16]. The quantitative evidence of acetylcholine production by *Lactiplantibacillus plantarum* and strategies to enhance its synthesis remain largely unexplored. Therefore, enhancing acetylcholine production through probiotic-based systems, such as *Lactiplantibacillus plantarum*, holds potential for improving cognitive function. Optimizing the production of acetylcholine through fermentation can lead to more effective probiotic formulations, which may provide an accessible and natural means to modulate the gut-brain axis and mitigate cognitive decline. Given that acetylcholine is also implicated in a variety of physiological processes, including muscle contraction and autonomic functions, increasing its production via biotechnological optimization may have broad applications in both therapeutic and preventative healthcare strategies.

Optimizing medium compositions and fermentation conditions is commonly done in biotechnology using design of experiments (DOE) [17, 18]. Statistical optimization enables the screening of numerous experimental factors and illustrates the significance of each factor [19, 20]. This optimization process includes four main stages: design of the experiment, conducting the planned experiments, predicting the best possible outcome, and validating the model [21]. The Taguchi design methodology is a concise and efficient experimental approach that employs orthogonal arrays to screen and optimize experimental conditions by identifying significant influencing variables with minimal experimental runs [22]. This design methodology facilitates the rapid and precise evaluation of the individual factors exhibiting significant main effects, and enables the selection of the optimal combination of factors to achieve the desired conditions [23]. This approach includes studying multiple variables through a limited number of experiments. While more advanced models such as response surface methodology or neural networks are required for exploring higher-order interactions, the Taguchi method remains highly valuable for initial parameter enhancement and rapid performance tuning. The Taguchi design methodology has been employed to optimize various metabolites produced by *Lactiplantibacillus* spp. This includes lipase production from co-culture of *Lactiplantibacillus brevis* and *Lactiplantibacillus plantarum* [24], as well as lactic acid production by *Lactiplantibacillus amylophilus* GV6 [25], *Lactiplantibacillus amylovorus* NRRL B-4542 [26], and *Lactiplantibacillus delbrueckii* NCIM 2025 [27]. To our knowledge, this method has not been applied to optimize acetylcholine production by *Lactiplantibacillus* sp.

Recently, Song et al. [28] reported the effects of *Lactiplantibacillus plantarum* DP 189 administration in alleviating cognitive deficits of Alzheimer’s disease in D-galactose-induced mice. Hu et al. [29] have used layer-by-layer encapsulation technology to encapsulate *Lactiplantibacillus plantarum* to improve their bioactivity and viability that are often compromised as they pass through the gastrointestinal tract in Alzheimer’s disease mice. Di Salvo et al. [30] reported that the oral intake of *Lactiplantibacillus plantarum* HEAL9 prevented cognitive decline in the prodromal phases of Alzheimer’s disease by modulating the signals between microbiota, gut, inflammasome, and brain in senescence-accelerated mouse prone 8. Hsiao et al. [31] have used *Lactiplantibacillus plantarum* encapsulated in okra polysaccharides in Alzheimer’s disease mice. The administration of *Lactiplantibacillus plantarum* markedly elevated their gut abundance and increased the level of short chain fatty acids. Huang et al. [32] reported that the supplementation of *Lactiplantibacillus plantarum* PS 128 prevented cognitive dysfunction induced by streptozotocin injection in Alzheimer’s disease mice by regulating the levels of glycogen synthase kinase 3 beta activity, propionic acid, and gliosis. Shamsipour et al. [33, 34] studied the effects of combined therapy including exercise training and 8 weeks administration of *Lactiplantibacillus plantarum* and *Bifidobacterium bifidum* on acetylcholine, neurotoxicity of Aβ, and spatial learning in Alzheimer disease rats. This combined therapy significantly increased acetylcholine, reduced the number of dead cells in the rats’ brains, and increased the time in Morris water maze test. Nimgampalle et al. [35] reported the administration of *Lactiplantibacillus plantarum* MTCC 1325 for 60 days, ameliorated cognition dysfunction and restored the level of acetylcholine and the histopathological features in Alzheimer disease albino rats. Mallikarjuna et al. [36] reported that *Lactiplantibacillus plantarum* MTCC 1325 delayed neurodegeneration in Alzheimer’s disease-induced rat brain by reverting all the constituents of ATPases system within 30 days. In a previous study, we investigated the characteristics of *Lactiplantibacillus plantarum* AM2 as a new probiotic strain and evaluated its effect in the prevention of cognitive deficits of Alzheimer’s disease in D-galactose-induced rats. Enhancing biomass production of *Lactiplantibacillus plantarum* AM2 can streamline the recovery process and lower production costs. Moreover, greater biomass yields can also shorten fermentation time, decrease wastewater generation, and expedite downstream processing. A pivotal study provided the earliest evidence of acetylcholine production by a strain of *Lactobacillus plantarum* [37]. Our study builds on this foundational observation by using a new *Lactiplantibacillus plantarum* strain AM2 for acetylcholine production and screen key fermentation factors that influence its biosynthesis. The aim of this study is to screen and optimize key fermentation factors affecting acetylcholine and biomass production by *Lactiplantibacillus plantarum* AM2 using the Taguchi array design. To our knowledge, this is the first systematic attempt to enhance acetylcholine biosynthesis by *Lactiplantibacillus plantarum* through controlled fermentation strategies. These findings may contribute to the development of probiotics targeting the gut-brain axis. This optimization also sheds light on the functional niche of AM2 strain when used as symbiotic. By adjusting the growth factors, the strain’s ability to produce acetylcholine will be maximized within the human gut microbiome hence, modulating the gut-brain axis, and offering therapeutic benefits for degenerative neurological condition. While other robust designs such as response surface methodology (RSM) or central composite design (CCD) are widely used for optimization, the Taguchi design was selected because of its simplicity, efficiency in minimizing the number of experiments, and its ability to handle complex interactions between multiple factors with relatively fewer experimental runs.

## Materials and methods

### Microorganism

The bacterium used throughout the current study was *Lactiplantibacillus plantarum* AM2 (EMCCN 4080).

### Media and chemicals

The culture media used throughout the present work were prepared according to the manufacturer’s instructions. The following media (g/l) were used. *De man, Rogosa and Sharpe medium (MRS) broth (Merck KGaA, Darmstadt, Germany)*: peptone from casein, 10.0; beef extract, 8.0; yeast extract, 4.0; D (+)-glucose, 20.0; dipotassium hydrogen phosphate, 2.0; tween 80, 1.0; di-ammonium hydrogen citrate, 2.0; sodium acetate, 5.0; magnesium sulfate, 0.2; and manganese sulfate, 0.04. The medium was supplemented with 25 mg choline (HiMedia, India)/l of medium as a precursor for acetylcholine (ACh) production [37].

### Seed inoculum

A loopful of *Lactiplantibacillus plantarum* AM2 pure culture was inoculated onto the surface of MRS agar and was then incubated at 37 °C under anaerobic conditions for 24 h. A seed culture of 10^9^ cfu/ml was prepared by adjusting the optical density (OD) using spectrophotometer (T80 + UV/VIS PG instrument LTD, UK) and 1.0% inoculum size (IS) was used throughout the experiments.

### Determination of acetylcholine

To promote ACh biosynthesis, the culture medium was supplemented with 25 mg/l choline chloride (HiMedia, India) as a biosynthetic precursor. Cell free supernatants of *Lactiplantibacillus plantarum* AM2 were collected and ACh was measured quantitatively *in-vitro* using competitive inhibition enzyme immunoassay kit. Briefly, in a microplate a competitive inhibition reaction was set between unlabeled ACh in standards or control or cell free supernatant (50 μl) and biotin labeled ACh (50 μl) with the precoated antibody specific to ACh. After incubation at 37 °C for 1 h, the unbound conjugate was washed off 3 times using 350 μl washing solution. Next, an aliquot of 100 μl of avidin conjugated to horseradish peroxidase (HRP) was added to each microplate well and incubated at 37 °C for 30 min. The washing process was repeated for 5 times using 350 μl washing solution. Next, an aliquot of 90 μl of substrate solution was added to each microplate well and incubated at 37 °C for 20 min. After incubation, 50 μl of stop solution was added to each microplate well, then the intensity of the developed color which is reverse proportional to the concentration of ACh in the samples was measured using a plate reader (Microlisa—Micro Lab Instruments, India) at 450 nm. A standard curve ranged from 12.35 to 100 pg/ml was conducted with the log of ACh concentration on the y-axis and absorbance on the x-axis. The average of triplicate readings for each standard, control, and samples was calculated. No extraction or purification step was conducted prior to measurement; thus, the values reported reflect the total ACh concentration present in the culture broth post-fermentation. In cases where samples were diluted, the measured value was multiplied by the dilution factor.

### Determination of Lactiplantibacillus plantarum AM2 growth

The biomass of *Lactiplantibacillus plantarum* AM2 was determined by using dry weight measurement. Briefly, 7 mL of samples taken from the fermentation flask was centrifuged at 4000 rpm for 15 min. The supernatant was removed, and cell pellets were dried at 60 °C for 24 h. The cell pellets were weighed on analytical balance (Sartorius LE244E, Germany) to obtain dry biomass weight. Optical density was measured by using a spectrophotometer (T80 + UV/VIS PG instrument LTD, UK) at 600 nm. The viable cell counts of *Lactiplantibacillus plantarum* AM2 were determined by pour plate method [38].

### Optimization of medium composition and fermentation conditions for maximum acetylcholine and biomass production by Lactiplantibacillus plantarum AM2

Optimization methodology adopted in the current study was divided into four sequentially interconnected phases viz., design of experiments, sub-merged fermentation experiments, analysis of experimental results and prediction of optimum settings, and validation of results.

#### Design of experiments (DOE)

The Taguchi DOE was used as a screening and optimization method to investigate the critical fermentation factors namely, beef extract (g/l), peptone (g/l), yeast extract (g/l), glucose (g/l), pH, agitation rate (rpm), and inoculation size % (v/v) that have an influence on ACh and biomass production while the other constituents of MRS broth were kept constant. In the next step, matrix was designed with the appropriate orthogonal array (OA) for the selected factors and their levels. In the present study, 12 experimental trials (OA L12) were selected for above controlled factors with two levels of factor variation (Table 1). The L12 orthogonal array is particularly useful when dealing with multiple dependent variables. It also ensures that the effect of each factor can be studied without requiring an excessive number of experiments, making the process cost-effective and time-efficient.

**Table 1:** Experimental setup using an L-12 orthogonal array for studying the effects of the selected factors on ACh and biomass production

Trial	Beef extract (g/l)	Peptone (g/l)	Yeast extract (g/l)	Glucose (g/l)	pH	Agitation rate (rpm)	Inoculation size% (v/v)
1	1	1	1	1	1	1	1
2	1	1	1	1	1	2	2
3	1	1	2	2	2	1	1
4	1	2	1	2	2	1	2
5	1	2	2	1	2	2	1
6	1	2	2	2	1	2	2
7	2	1	2	2	1	1	2
8	2	1	2	1	2	2	2
9	2	1	1	2	2	2	1
10	2	2	2	1	1	1	1
11	2	2	1	2	1	2	1
12	2	2	1	1	2	1	2

#### Sub-merged fermentation

Sub-merged fermentation experiments were performed for ACh and biomass production with *Lactiplantibacillus plantarum* AM2 employing the selected 12 experimental trails (Table 1). All experiments were conducted in 100 ml Erlenmeyer flasks each containing 20 ml fermentation medium. The flasks were inoculated with the levels of inoculum indicated in Table 1 and were incubated at 37 °C for 48 h. After incubation, ACh concentration (pg/ml) and biomass (g/l) were determined. The results presented in the current experiment are the average of three individual determinations.

#### Analysis of experimental results and prediction of optimum settings

The influence of individual factors on ACh (pg/ml) and biomass (g/l) production and their performance at optimum conditions using Taguchi approach were analyzed by Statistica 10 software. In Taguchi’s method, quality is measured by the deviation of a characteristic from its target value and a loss function $[L\left(y\right)]$ is estimated for the deviation [39] as $L\left(y\right)={K \times (y -m)}^{2}$, where k denotes the proportionality constant, *m* represents the target value and *y* is the experimental value obtained for each trial. In case of bigger and better quality characteristics, the loss function can be written as $L\left(y\right)= K \times \frac{1}{{y}^{2}}$ and the expected loss function can be represented by $E[L\left(y\right)]= K \times \frac{1}{{y}^{2}}$ where $E(\frac{1}{{y}^{2}})$ can be estimated from *n* number of samples as $\frac{\sum_{i=1}^{n}\frac{1}{{y}_{i}^{2}}}{n}$.

#### Validation of results

To validate the optimized methodology, the fermentation experiments were performed in triplicates for ACh (pg/ml) and biomass (g/l) production under the optimized and basal culture conditions.

## Results

### Optimization of medium composition and fermentation conditions by Taguchi array design for maximum acetylcholine and biomass production by Lactiplantibacillus plantarum AM2

**Fig. 1:**
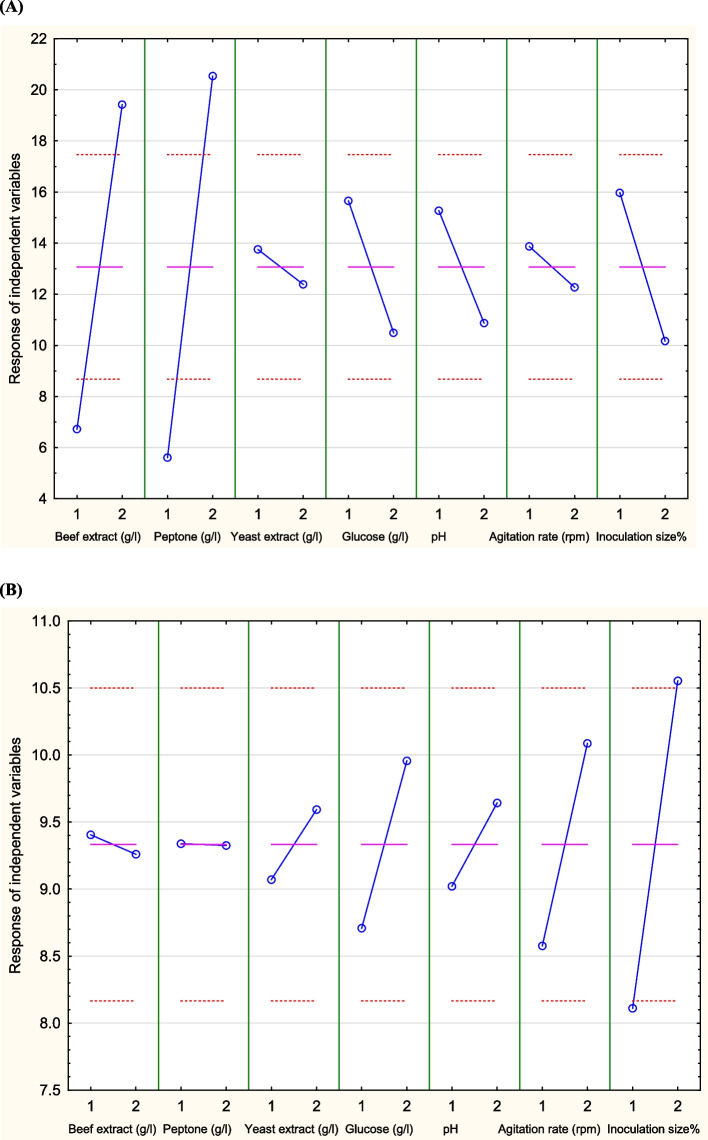
Response graph illustrating the effects of different factors on ACh production (pg/ml) (**A**) and biomass production (g/l) (**B**) by *Lactiplantibacillus plantarum* AM2

Seven factors that have great effect on ACh and biomass production by *Lactiplantibacillus plantarum* AM2, were selected [2]. Optimization of medium composition and fermentation conditions was carried out using Taguchi array design with an orthogonal array (OA) L12. The main responses of factors on the production of ACh (pg/ml) and biomass (g/l) by *Lactiplantibacillus plantarum* AM2 are presented in Table 2 and Fig. 1. The highest ACh concentration (395 pg/ml) was observed in run 10 where the levels of beef extract (g/l), peptone (g/l), yeast extract (g/l), glucose (g/l), pH, agitation rate (rpm), and inoculation size % (v/v) in the order of 2, 2, 2, 1, 1, 1, and 1, respectively. Overall, higher levels of beef extract and peptone were associated with increased ACh production, while yeast extract had a marginal or negative effect. On the other hand, the highest biomass concentration (13.14 g/l) was obtained in run 6 where the levels of beef extract (g/l), peptone (g/l), yeast extract (g/l), glucose (g/l), pH, agitation rate (rpm), and inoculation size % (v/v) in the order of 1, 2, 2, 2, 1, 2, and 2, respectively.

**Table 2:** Main response values of factors at assigned levels affecting ACh production (pg/ml) and biomass production (g/l)

Run No	Beef extract (g/l)	Peptone (g/l)	Yeast extract (g/l)	Glucose (g/l)	pH	Agitation rate (rpm)	Inoculation size% (v/v)	Main response	
								**ACh concentration (pg//ml)**	**Biomass (g/l)**
1	8	10	5	20	5.7	0	1	77	6.00
2	8	10	5	20	5.7	150	4	78	10.43
3	8	10	20	35	6.6	0	1	64	9.54
4	8	40	5	35	6.6	0	4	83	9.18
5	8	40	20	20	6.6	150	1	151	8.14
6	8	40	20	35	5.7	150	4	132	13.14
7	11	10	20	35	5.7	0	4	79	9.57
8	11	10	20	20	6.6	150	4	104	10.50
9	11	10	5	35	6.6	150	1	116	10.00
10	11	40	20	20	5.7	0	1	395	6.68
11	11	40	5	35	5.7	150	1	337	8.32
12	11	40	5	20	6.6	0	4	316	10.50

**Fig. 2:**
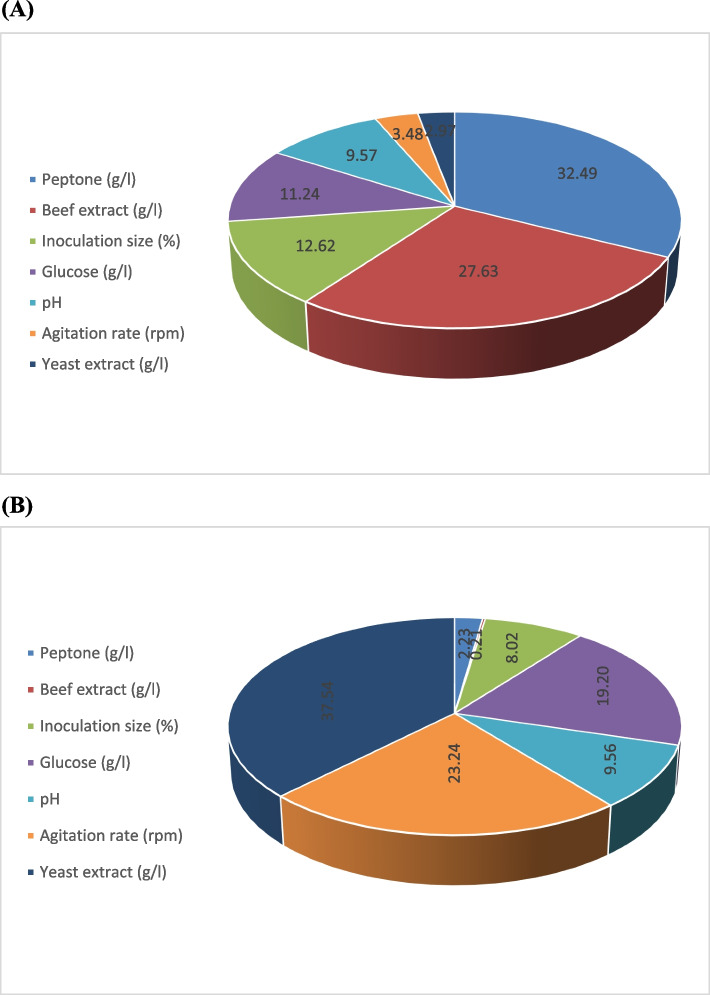
Percent contribution of different variables to ACh production (pg/ml) (**A**) and biomass production (g/l) (**B**) by *Lactiplantibacillus plantarum* AM2

The statistical analysis of the Taguchi experimental results (Table 3) provides insights into the significance of each factor in determining the production of ACh (pg/ml) and biomass (g/l), the *P*-value for each response was determined at 95% or higher confidence level. The data indicated that beef extract (g/l) and peptone (g/l) significantly influenced ACh production by AM2 strain, as indicated by their *P*-values (< 0.05), while yeast extract (g/l), glucose (g/l), pH, agitation rate (rpm), and inoculation size % (v/v) had insignificant effects. On the other hand for biomass production by the AM2 strain, the most influential factor was inoculation size% (v/v), which had the highest *t*-value and statistical significance. Whereas, beef extract (g/l), peptone (g/l), yeast extract (g/l), glucose (g/l), pH, and agitation rate (rpm) had insignificant effects. Interestingly, glucose showed a positive trend in influencing biomass yield, although it was not statistically significant (*P* = 0.2047). As illustrated in Fig. 2, the contribution percentage of each independent variable to ACh (pg/ml) production was in the order of peptone (g/l), beef extract (g/l), inoculation size % (v/v), glucose (g/l), pH, agitation rate (rpm), and yeast extract (g/l). While the percent contribution of each independent variable to cell growth (g/l) was in the order of yeast extract (g/l), agitation rate (rpm), glucose (g/l), pH, inoculation size % (v/v), peptone (g/l), and beef extract (g/l). Furthermore, the percent contribution analysis reveals that peptone (32.49%) and beef extract (27.63%) are the most significant contributors to ACh production, reinforcing their critical role in enhancing ACh yield. On the other hand, biomass production is primarily influenced by yeast extract (37.54%), agitation rate (23.24%), and glucose concentration (19.2%). These findings provide insights into the metabolic requirements of *Lactiplantibacillus plantarum* AM2.

**Table 3:** Statistical analysis of Taguchi experimental results, including coefficient values, *t*-test results, and *P*-values

Variable	ACh concentration (pg/ml)				Biomass (g/l)			
	**Coefficient**	**Standard error**	**Computed *t*-value**	***P*-value**	**Coefficient**	**Standard error**	**Computed *t*-value**	***P*-value**
Intercept	21.5	124.41	0.17	0.87119	0.052	3.298	0.016	0.988254
Beef extract (g/l)	127	31.10	4.08	0.01505	−0.145	0.825	−0.176	0.868978
Peptone (g/l)	149.33	31.10	4.80	0.00864	−0.013	0.825	−0.016	0.987875
Yeast extract (g/l)	−13.66	31.10	−0.44	0.68307	0.522	0.825	0.633	0.561384
Glucose (g/l)	−51.66	31.10	−1.66	0.17202	1.248	0.825	1.514	0.204669
pH	−44	31.10	−1.41	0.23008	0.622	0.825	0.754	0.49291
Agitation rate (rpm)	−16	31.10	−0.51	0.63407	1.512	0.825	1.833	0.140741
Inoculation size % (v/v)	−58	31.10	−1.86	0.13565	2.442	0.825	2.961	0.041528

^*^Significant (*P*-value ≤ 0.05)

First-order regression was performed using multiple regression analysis in order to obtain a regression equation that describes the correlations between the 7 factors. The obtained models can be observed by the following equations:

$$\text{Y}1 = 21.5 + 127\text{X}1 + 149.3\text{X}2 - 13.66\text{X}3 - 51.66\text{X}4 - 44\text{X}5 - 16\text{X}6 - 58\text{X}7$$

where Y1 is the predicted response or ACh concentration (pg/ml) and X1, X2, X3, X4, X5, X6, and X7 are the coded values of the test variables beef extract (g/l), peptone (g/l), yeast extract (g/l), glucose (g/l), pH, agitation rate (rpm), and inoculation size % (v/v), respectively.

$$\text{Y}2 = 0.05 - 0.145\text{X}1- 0.013\text{X}2 + 0.52\text{X}3 + 1.25\text{X}4 + 0.622\text{X}5 + 1.51\text{X}6 + 2.44\text{X}7$$

where Y2 is the predicted response or biomass concentration (g/l) and X1, X2, X3, X4, X5, X6, and X7 are the coded values of the test variables beef extract (g/l), peptone (g/l), yeast extract (g/l), glucose (g/l), pH, agitation rate (rpm), and inoculation size % (v/v), respectively.

The model predicted that the optimum levels of independent variables for maximum ACh production were beef extract, 11 g/l; peptone, 40 g/l; yeast extract, 5 g/l; glucose, 20 g/l; pH, 5.7; agitation rate, 0 rpm; and inoculation size, 1% (v/v) (in the order of 2, 2, 1, 1, 1, 1 and 1). On the other hand, the optimal conditions for maximum biomass production were beef extract, 8 g/l; peptone, 10 g/l; yeast extract, 20 g/l; glucose, 35 g/l; pH, 6.6; agitation rate, 150 rpm; and inoculation size, 4% (v/v) (in the order of 1, 1, 2, 2, 2, 2 and 2).

The ANOVA results (Tables 4, 5 and 6) confirm the statistical significance of the regression models, the results of ACh production model was significant (*P* < 0.05), the model F value of 6.917 was representative of significance, R^2^ was equal to 0.9236, which is in acceptable agreement with the adjusted R^2^ value of 0.7901. This indicates that the selected factors played an important role in determining ACh levels. On the other hand, the results of biomass production model was also significant (*P* < 0.05), the model F value of 7.202 was representative of significance, R^2^ was equal to 0.9739 which in acceptable agreement with the adjusted R^2^ value of 0.843. This reinforces the impact of inoculation size on biomass yield. These results validate the experimental design and suggest that the optimized conditions can effectively enhance ACh and biomass production. The ANOVA results also confirm the statistical significance of the models for both ACh and biomass production, with *P*-values below 0.05. The relatively high F-values suggest a strong influence of the tested variables on the experimental outcomes. Regression analysis indicates that the models fit well with ACh and biomass production, respectively. This demonstrates that the selected factors adequately explain the variability in the data.

**Table 4:** Analysis of variance (ANOVA) results for ACh production (pg/ml) by *Lactiplantibacillus plantarum* AM2 based on the Taguchi experiment

	***Df***	***SS***	***MS***	***F***	***P-value***
Regression	7	140,525	20,075	6.917047	0.040271019
Residual	4	11,609	2902.25		
Total	11	152,134			

^*^Significant (*P*-value ≤ 0.05)

**Table 5:** Analysis of variance (ANOVA) results for biomass production (g/l) by *Lactiplantibacillus plantarum* AM2 based on the Taguchi experiment

	***Df***	***SS***	***MS***	***F***	***P-value***
Regression	7	31.45505	4.493579	7.202271	0.023239927
Residual	4	8.161717	2.040429		
Total	11	39.61677			

^*^Significant (*P*-value ≤ 0.05)

**Table 6:** Regression statistics showing the impact of variables on ACh (pg/ml) and biomass (g/l) production by *Lactiplantibacillus plantarum* AM2

Regression statistics for variables affecting ACh production				Regression statistics for variables affecting biomass production			
**R**^**2**^	**Adjusted R**^**2**^	**Standard error**	**Observations**	**R**^**2**^	**Adjusted R**^**2**^	**Standard error**	**Observations**
0.9236922	0.7901	53.872	12	0.973983	0.843345	1.42843	12

**Fig. 3:**
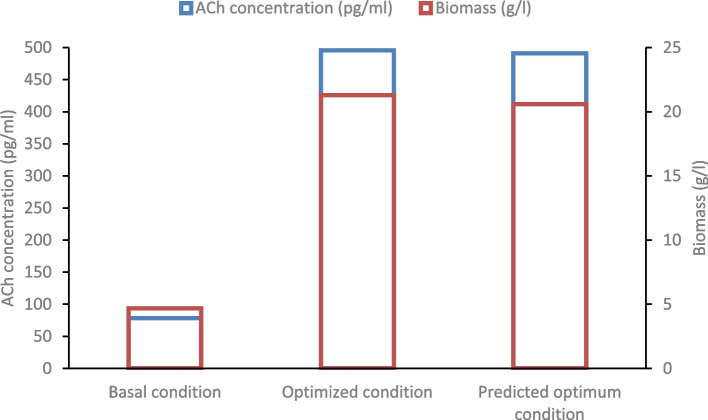
Comparative analysis of ACh (pg/ml) and biomass (g/l) production under three conditions: optimized, predicted, and basal, for *Lactiplantibacillus plantarum* AM2

In order to verify the predicted optimum levels of factors affecting on ACh (pg/ml) and biomass (g/l) production by *Lactiplantibacillus plantarum* AM2, a validation experiment was conducted. As shown in Fig. 3, a very good agreement is observed between the predicted ACh production (490.83 pg/ml) and experimental ACh production (495.8 pg/ml). Likewise, the predicted biomass production (20.58 g/l) and the observed biomass production (21.3 g/l). Significant improvement of 417.44 pg/ml ACh and 16.63 g/l biomass were achieved under the optimum medium, compared with ACh and biomass levels achieved under the basal conditions. Consequently, ACh and biomass production increased 6.32 and 4.56 times greater than the basal condition through the Taguchi method.

## Discussion

In a previous study, we explored the characteristics of *Lactiplantibacillus plantarum* AM2 as a novel probiotic strain and assessed its potential in preventing cognitive deficits associated with Alzheimer’s disease in D-galactose-induced rats. Enhancing the biomass production of *Lactiplantibacillus plantarum* AM2 offers significant advantages, such as simplifying the recovery process, reducing production costs, shortening fermentation times, minimizing wastewater generation, and accelerating downstream processing. In the present study, we optimized the fermentation conditions to enhance biomass production, paving the way for industrial applications. This is also the first study to focus on optimizing fermentation conditions to increase ACh production by *Lactiplantibacillus plantarum* AM2. This optimization provides insight into the functional niche of the AM2 strain when used as a symbiotic. By fine-tuning growth factors, the strain’s ACh production can be maximized within the human gut microbiome, thereby modulating the gut-brain axis and offering therapeutic potential for degenerative neurological conditions.

Taguchi design of experiment is based on orthogonal arrays that allows simultaneous assessment of the combined effects of independent variables and estimates their optimal levels [40–46]. Seven factors viz., beef extract (g/l), peptone (g/l), yeast extract (g/l), glucose (g/l), pH, agitation rate (rpm), and inoculation size % (v/v) which have the most prominent influence on ACh (pg/ml) and biomass (g/l) production by *Lactiplantibacillus plantarum* AM2 have been selected. Although the interaction between these factors is complex, applying the Taguchi method renders it possible using less number of experiments. The Taguchi method was used to determine the optimal levels for the best conditions on ACh (pg/ml) and biomass (g/l) production by *Lactiplantibacillus plantarum* AM2. In order to achieve this purpose, the quality characteristic the “larger the better” was selected for processing the obtained experimental data. The chosen levels of beef extract, peptone, yeast extract, glucose, pH, agitation rate, and inoculation size were systematically varied to evaluate their combined impact. This design ensures a comprehensive exploration of factor interactions while minimizing the number of experimental trials required.

The optimum levels of independent variables revealed by the obtained model for maximum ACh production (pg/ml) by *Lactiplantibacillus plantarum* AM2 were as follows: beef extract, 11 g/l; peptone, 40 g/l; yeast extract, 5 g/l; glucose, 20 g/l; pH, 5.7; agitation rate, 0 rpm; and inoculation size, 1% (v/v). To the best of our knowledge, no study has been conducted to investigate the enhancement of ACh production by *Lactiplantibacillus* sp. through the Taguchi approach. However, Hwang et al. [47] reported the utilization of the Taguchi orthogonal array and the Box-Behnken design to optimize medium formulation for improved biomass yield of *Lactiplantibacillus plantarum* Pi06. On the other hand, the optimal conditions for maximum biomass production (g/l) by the AM2 strain were as follows: beef extract, 8 g/l; peptone, 10 g/l; yeast extract, 20 g/l; glucose, 35 g/l; pH, 6.6; agitation rate, 150 rpm; and inoculation size, 4% (v/v). These findings align with reports from other researchers studying growth optimization of different *Lactiplantibacillus plantarum* strains. Yoo et al. [2] reported that the optimal concentrations of medium components for the growth of *Lactiplantibacillus plantarum* JNU 2116 using response surface methodology were determined to be 0.213% soy-peptone, 1.232% glucose, 1.97% yeast extract, and 0.08% magnesium sulfate. Gokmen et al. [48] stated that the specific growth rate of *Lactiplantibacillus plantarum* BG24 in the original MRS broth (pH 5.7) was 0.416 h^−1^, with a doubling time of 1.67 h and biomass productivity of 0.14 g/l/h. In contrast, when cultured in MRS broth (pH 6.5) enriched with 5 g/l yeast extract, the values increased to 0.483 h^−1^ for the growth rate, 1.43 h for the doubling time, and 0.17 g/l/h for biomass productivity. Noori et al. [49] demonstrated that the optimal conditions for the growth of *Lactiplantibacillus plantarum* T5 JQ301796.1 in a lab-scale fermentor using response surface methodology were found to be 25.96 g/l glucose, 1.82% yeast extract, a pH of 7.26, and stirring at 40 rpm, with the temperature maintained between 37–40 °C. Under these conditions, the maximum viable cell count achieved in batch fermentation was 1.25 × 10^10^ cfu/ml. Choi et al. [50] indicated that the optimal medium composition determined by response surface methodology consisted of 31.29 g/l maltose, 30.27 g/l yeast extract, 39.43 g/l soytone, 5 g/l sodium acetate, 2 g/l K_2_HPO_4_, 1 g/l tween 80, 0.1 g/l MgSO_4_.7H_2_O, and 0.05 g/l MnSO_4_.H_2_O, with a predicted maximum biomass of 3.951 g/l. Under these optimized conditions, the biomass of *Lactiplantibacillus plantarum* 200655 reached 3.845 g/l, closely matching the predicted value and 1.58 times higher than the biomass obtained with the unoptimized medium (2.429 g/l). Additionally, biomass increased further to 4.505 g/l under the optimized cultivation conditions. Hwang et al. [47] revealed that the optimized medium for the growth of *Lactobacillus plantarum* Pi06 using Taguchi design included 35 g/l glucose, 35 g/l yeast extract, and 40 ml/l corn steep liquor. When compared to the initial medium, the biomass yield increased from 4.31 to 8.94 g/l, representing an improvement of about 107%.

The R^2^ and adjusted R^2^ statistic clearly demonstrated that the model as fitted explains 92.37% and 97.39% of the variability in the parameters of ACh (pg/ml) and biomass (g/l) production, respectively. The fitted model generated by the Taguchi design provided a very close approximation to the real system which was verified by conducting a validation experiment. The production of ACh (pg/ml) and biomass (g/l) by *Lactiplantibacillus plantarum* AM2 was raised, respectively, by 6.32 and 4.56 fold increase when compared to the basal conditions applied before optimization.

Future studies could scale up this optimized process for industrial applications. This may involve implementing a controlled bioreactor system with automated pH, agitation, and aeration adjustments to maintain optimal conditions at larger volumes. Alternative cost-efficient nitrogen sources with similar effects could also be explored. The influence of inoculum size suggests that optimizing seed culture preparation is crucial for high biomass yield and ACh productivity in industrial fermentation. Efficient extraction and purification techniques must be developed while maintaining cost efficiency. While the Taguchi design effectively optimized significant variables with a reduced number of experiments, future work could extend this study using response surface methodology, central composite design or artificial neural networks for deeper optimization of higher order interaction between factors [51].

## Conclusion

This study successfully optimized the fermentation conditions and medium composition for maximum ACh and biomass production by *Lactiplantibacillus plantarum* AM2 using the Taguchi array design. The results demonstrated significant improvements in both ACh and biomass yields, with 6.32 fold and 4.56 fold enhancements, respectively, compared to the baseline conditions. The findings of this research provide valuable insights into the metabolic potential and functional niche of *Lactiplantibacillus plantarum* AM2, paving the way for further studies on its therapeutic and industrial applications. Despite the success of the optimization process, certain limitations should be acknowledged. First, the study focused solely on the Taguchi method, which, while efficient, does not explore higher-order interactions among variables as effectively as other optimization strategies like artificial neural networks. Additionally, the fermentation experiments were conducted at a laboratory scale, and the results may not be directly translatable to industrial-scale bioreactors without further process validation. Future work may focus on scaling up these optimized conditions, investigating the strain’s performance in diverse environmental setups, and exploring its in vivo synergistic effects in symbiotic formulations.

## Data Availability

The datasets supporting the conclusions of this article are included within the article.
